# AI-Enhanced Analysis of Built Environment Imagery and Neighborhood Obesity in US Cities

**DOI:** 10.1001/jamanetworkopen.2025.34612

**Published:** 2025-09-30

**Authors:** Zhuo Chen, Tong Zhang, Jean-Eudes Dazard, Sai Rahul Ponnana, Weichuan Dong, Skanda Moorthy, Santosh Kumar Sirasapalli, Haitham Khraishah, Salil Deo, Sanjay Rajagopalan, Sadeer Al-Kindi

**Affiliations:** 1 School of Medicine, Case Western Reserve University, Cleveland, Ohio; 2 Harrington Heart and Vascular Institute, University Hospitals, Cleveland, Ohio; 3 Houston Methodist DeBakey Heart and Vascular Center, Houston, Texas

## Abstract

**Importance:**

Obesity prevalence is a significant public health issue, particularly in urban areas. While social factors are known risks, using artificial intelligence (AI) to scalably assess the association of the built environment with obesity prevalence is an emerging area critical for targeted interventions.

**Objective:**

To investigate whether AI analysis of satellite and street view imagery is associated with improved estimates of neighborhood obesity prevalence beyond conventional demographic and socioeconomic (DSE) and social determinants of health (SDOH) factors.

**Design, Setting, and Participants:**

This cross-sectional study used an AI-enhanced modeling framework. Data from the 2023 Centers for Disease Control and Prevention (CDC) PLACES dataset and 2019 American Community Survey were linked to geospatial imagery retrieved between May and July 2024 for US Census tracts within 94 of the 100 most populous US cities (6 were excluded due to missing obesity data). The unit of analysis was the census tract. Data included more than 94 000 Google satellite images and 670 000 Street View images, which, along with obesity prevalence, DSE, and SDOH factors, were all linked at the census tract level. The analysis was conducted from September 2024 to May 2025.

**Exposures:**

Built environment features were extracted from satellite and street view imagery using convolutional neural networks.

**Main Outcomes and Measures:**

Crude obesity prevalence at the census tract level (adults aged ≥18 years with body mass index ≥30.0) was obtained from the 2023 CDC PLACES dataset.

**Results:**

The study included 14 413 census tracts (median [IQR] resident age, 35 [32-40] years; median [IQR], 51.1% [48.7%-53.8%] female) with a median (IQR) obesity prevalence of 32.4% (26.6%-38.8%). At the census tract level, the median (IQR) population was 3861 (2701-5210) residents, with a median (IQR) annual household income of $56 042 ($38 494-$80 859). The study processed 94 498 satellite and 670 860 street view images. A linear mixed-effects model including DSE, SDOH, satellite images, and street view imagery features explained 92.6% of the variance in obesity prevalence (conditional *R*^2^ = 0.926, including random effects). Adding image-derived features to a model with DSE and SDOH covariates was associated with an increase in the variance explained by fixed effects, with an increase in the marginal *R*^2^ from 0.632 to 0.745 (χ^2^ = 1303.4; *P* < .001).

**Conclusions and Relevance:**

In this study, integrating AI-derived built environment features from geospatial imagery was associated with enhanced ability to explain and estimate neighborhood-level obesity prevalence beyond conventional DSE and SDOH factors.

## Key Points

**Question:**

Is artificial intelligence–enhanced geospatial image analysis of built environment features associated with improved estimates of neighborhood obesity prevalence in US cities beyond traditional factors?

**Findings:**

This cross-sectional study of 14 413 census tracts within 100 US cities found that integrating geospatial image features from more than 94 000 satellite and more than 670 000 street view images with traditional factors in a linear mixed-effects model was associated with improved obesity prevalence variance explanation.

**Meaning:**

In this study, artificial intelligence–driven geospatial image analysis was associated with enhanced obesity prevalence estimation, which may inform targeted public health and urban planning interventions.

## Teaser

This cross-sectional study investigates the association of artificial intelligence (AI)–enhanced analysis of geospatial built environment images with estimates of neighborhood obesity prevalence.

## Introduction

The escalating prevalence of obesity constitutes a significant and growing public health challenge globally and within the US.^1,2,3^ While historical data indicate a substantial increase in obesity prevalence in the US, from 30.5% in 2000 to 42.4% in 2018,^4^ recent projections suggest a concerning future trajectory.^5^ Absent immediate and effective interventions, it is estimated that nearly 260 million individuals in the US will be living with overweight or obesity by 2050.^5^ An expanding obesity epidemic is clinically significant due to its strong association with a range of obesity-related complications, including cancers, type 2 diabetes, and cardiovascular diseases. These associations further contribute to a considerable public health burden through increased morbidity and premature mortality, with annual medical costs for obesity in the US estimated at nearly $173 billion in 2019 dollars.^6^

While conventional risk factors, such as healthy nutrition, energy metabolism, and lack of physical activity, are undoubtedly significant, a substantial body of evidence now recognizes the critical role of the built environment in shaping obesity prevalence.^7,8,9,10,11,12^ The design of cities and neighborhoods, encompassing buildings, transportation systems, and public spaces, directly influences risk factors, such as physical activity and dietary behaviors.^10,11,13,14^ Features, such as the availability of green spaces, pedestrian-friendly sidewalks, bike trails, and accessible exercise facilities, have been consistently associated with lower obesity prevalence and improved public health outcomes.^7,15,16^ Conversely, environments lacking these features may inadvertently promote sedentary lifestyles and unhealthy dietary patterns.^17^

Traditional assessments of the built environment often rely on surveys or secondary data, which can lack detail and consistency.^11,18^ In response to these limitations, geospatial imaging technologies, particularly satellite and street view imagery, offer a transformative approach to assess the built environment at scale.^15,19,20,21^ The increasing availability and high quality of geospatial images has made them a promising alternative to traditional survey and secondary data in measuring the large-scale built environment, especially with state-of-the-art deep learning algorithms.^22,23^ Deep neural networks, such as deep convolutional neural networks (CNNs), have been proven to be effective in computer vision tasks, such as object recognition, image classification, image segmentation, and image reconstruction.^24,25^

Inspired by the success of deep learning in analyzing satellite and street view images for tasks like economic prediction^26^ and socioeconomic status estimation,^27,28^ initial attempts have been made to apply these techniques to public health research, including cardiovascular diseases and^19,20,29,30^ brain disorders,^31^ as well as in the context of obesity.^21,32,33^ However, a holistic and interpretable artificial intelligence (AI)–driven approach to understanding the heterogeneity in neighborhood obesity prevalence is still lacking. In this study, we developed a novel, interpretable, AI-driven pipeline using satellite and street view imagery to assess the association of environmental factors with obesity. We hypothesized that image-derived features of the built environment would be associated with enhanced explanation of variance in neighborhood-level obesity prevalence beyond what is possible using traditional demographic and socioeconomic (DSE) and social determinants of health (SDOH) factors alone.

## Methods

**Figure 1.**
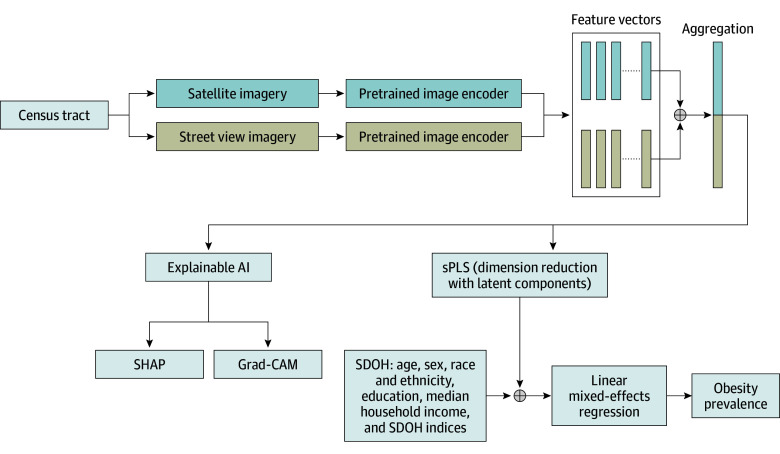
Study Framework Census tract–level satellite and street view images were processed using pretrained deep learning models (ResNet-50) to extract feature vectors, which were aggregated for each census tract. Feature importance and explainability were assessed using Shapley additive explanations (SHAP) values and gradient-weighted class activation mapping (grad-CAM) visualizations. A sparse partial least squares (sPLS) model was applied for dimension reduction, integrating social determinants of health (SDOH) before modeling obesity prevalence using linear mixed-effects regression. The final model evaluated the combined association of built environment features and SDOH with obesity prevalence across US cities. AI indicates artificial intelligence.

This cross-sectional study focused on census tracts within the 100 most populous US cities, excluding 6 cities in Florida due to missing obesity prevalence data in the 2023 Centers for Disease Control and Prevention (CDC) PLACES dataset (eFigure 1 in Supplement 1).^34^ The unit of analysis was the census tract. Figure 1 provides an overview of the modeling framework, including image processing, feature extraction, statistical modeling, and feature importance and visualization. The University Hospitals institutional review board exempted this study from review and the need for informed consent in accordance with 45 CFR §46 given that it relied exclusively on publicly available, aggregated data and was thus not considered human participants research. This study adhered to the Strengthening the Reporting of Observational Studies in Epidemiology (STROBE) reporting guideline.

### Data Sources

Data for this study were obtained from the 2023 CDC PLACES dataset at the census tract level. This dataset provides model-based estimates derived from the Behavioral Risk Factor Surveillance System (BRFSS), US Census Bureau, and American Community Survey (ACS). Specifically, the 2023 PLACES release uses BRFSS data from 2021 for 29 measures and 2020 for 7 measures collected biennially (all teeth lost, dental visits, mammograms, cervical cancer screening, colorectal cancer screening, core preventive services among older adults, and sleeping <7 hours), alongside Census Bureau 2010 population data and ACS 2015 to 2019 estimates.^34^

### Outcome Variable

The primary outcome was crude obesity prevalence at the census tract level, defined as the proportion of adults aged 18 years or older with a body mass index (calculated as weight in kilograms divided by height in meters squared) of 30.0 or greater. These estimates are generated by the CDC PLACES project using a multilevel regression and poststratification approach applied to BRFSS and ACS data^35^ based on self-reported weight and height.^36^

### Built Environment Feature Extraction and Preprocessing

Our primary exposures were built environment features extracted from Google satellite images (hereafter, *satellite images* [SIs]) and Street View (hereafter, *street view* [SV]) images for each census tract. We used a pretrained ResNet-50 CNN to generate a 4096-dimensional feature vector from each image. These image-level features were then aggregated to create a single representative built environment signature for each census tract. Full details regarding the image sampling protocol, CNN pretraining, and feature aggregation are available in the eMethods in Supplement 1.

### Covariates

We included a set of DSE and other SDOH covariates at the census tract level. DSE variables included 2019 ACS DSE factors, such as proportion male, proportion Black, median age, proportion Hispanic, proportion with education less than high school, and median household income. Race and ethnicity data were obtained from the ACS. Analyses were limited to Black and White populations for race and Hispanic populations for ethnicity because these groups represented the largest racial and ethnic populations across US census tracts and provided sufficient sample sizes for stable estimation. Other groups (American Indian or Alaska Native, Asian, Native Hawaiian or Other Pacific Islander, some other race, and 2 or more races) were not analyzed separately owing to small population sizes within census tracts, which limited statistical power and precluded reliable group-specific associations. We also incorporated established SDOH indices, including the social vulnerability index (SVI),^37^ social deprivation index (SDI),^38^ and area deprivation index.^39^

### Statistical Analysis

The dataset, stratified by city, was randomly divided into training (70%) and testing (30%) sets. A multistep modeling approach was implemented to evaluate associations of SI and SV features with obesity prevalence and the incremental contribution of SI and SV features to obesity prevalence beyond conventional risk factors, specifically DSE factors and SDOH indices. We first applied sparse partial least squares (SPLS) regression^40^ on the training set to reduce the dimensionality of SI and SV features while simultaneously selecting the most relevant components. Optimal hyperparameters for the number of components (*K*) and sparsity (η) were determined via 10-fold cross-validation to minimize estimation error (eMethods in Supplement 1). These SPLS components, alongside the original DSE and SDOH variables, were then incorporated as fixed effects into a linear mixed-effects model (LMEM).^41^ To account for potential clustering effects at the city level, city was included as a random intercept in the LMEM.

Next, we fitted a series of nested models and compared their performance and complexity. These models included variations using SI features only (model 1), SV features only (model 2), combined SI and SV features (model 3), DSE covariates only (model 4), DSE with SI and SV (model 5), SDOH covariates only (model 6), SDOH with SI and SV (model 7), DSE with SDOH (model 8), and DSE with SDOH and SI and SV (model 9). Model comparison was based on goodness of fit metrics, including Akaike information criterion (AIC), bayesian information criterion (BIC), and likelihood ratio tests (LRTs). Model performance was also assessed using marginal and conditional *R*^2^ values calculated according to the Nakagawa method for mixed models.^42^ Marginal *R*^2^ reflects the proportion of variance explained by fixed effects, while conditional *R*^2^ indicates the variance explained by fixed and random effects. The difference between conditional and marginal *R*^2^ values was interpreted as the proportion of variance attributable to the random effect of city.

To enhance model interpretability, we first trained an XGBoost software version 2.0.3 (XGBoost Contributors) model to assess the contribution of SI and SV features to obesity prevalence (eMethods in Supplement 1). Feature importance was assessed using total gain and Shapley additive explanations (SHAP) values.^43^ Based on the most influential features, gradient-weighted class activation mapping (grad-CAM)^44^ was then applied to visualize key image regions associated with model output. Grad-CAM overlays were generated by calculating gradients of the model output with respect to the final convolutional layer’s feature maps, finding means of these gradients to obtain channelwise weights, and producing a weighted activation map that was upsampled and overlaid on the original images.. A full workflow is provided in eFigure 2 in Supplement 1.

We processed images and implemented grad-CAM using Python software version 3.10.13 (Python Software Foundation) with PyTorch and fastai libraries. The machine learning modeling used the XGBoost library. LMEM and SPLS regression were performed in R statistical software version 4.4.2 (R Project for Statistical Computing) using lme4 and spls packages, respectively. Statistical significance of fixed effects in LMEM was assessed using Wald *t* tests, with a significance threshold of a 2-sided *P* < .05. Data were analyzed from September 2024 to May 2025.

## Results

### Descriptive Statistics of Demographic and Socioeconomic Characteristics

We analyzed 14 413 census tracts (median [IQR (range)] resident age, 35 [32-40 (6-76)] years; median [IQR] 51.1% [48.7%-53.8%] female and 48.9% [46.2%-51.3%] male; median [IQR], 9.5% [3.2%-33.7%] Black and 60.2% [31.6%-38.1%] White; median [IQR], 15.8% [6.2%-40.6%] Hispanic) across 94 US cities, from which we processed 94 498 SI and 670 860 SV images. Obesity prevalence varied widely across census tracts (eFigure 3 in Supplement 1), with a median (IQR [range]) of 32.4% (26.6%-38.8% [12.6%-61.1%]) of residents with obesity. The median (IQR [range]) annual household income was $56 042 ($38 494-$80 859 [$4129-$250 001]), and the median (IQR) census tract population was 3861 (2701-5210) residents. More details on demographics and socioeconomics of these tracts are provided in eTable 1 in Supplement 1.

### SPLS Regression Analysis of SI and SV Components

To evaluate the incremental benefits associated with SI and SV features beyond conventional risk factors in estimating obesity prevalence, we applied SPLS regression for dimension reduction and variable selection. Using cross-validation to optimize hyperparameters, we determined the optimal sparsity parameter (η) and number of components (*K*) for SI and SV features. For SV features, cross-validation results indicated that the optimal η was 0.4 and the optimal number of components (*K*) was 97 (eFigure 4 in Supplement 1). For SI features, the optimal η was determined to be 0.5, with *K* optimized at 71. These optimized hyperparameters were then used to fit final SPLS models for SI and SV, and their resulting components were incorporated into the LMEM alongside other DSE and SDOH variables. Projections of SI and SV using uniform manifold approximation and projection (eFigures 5 and 6 in Supplement 1) components revealed distinct clustering patterns associated with obesity prevalence across census tracts.

### Linear Mixed-Effects Model Results

Results of the LMEM analysis are summarized in the Table. Across models, the inclusion of geospatial imaging features (SI and SV) and DSE and SDOH indices was associated with improved explanation of obesity prevalence variance at the census tract level.

**Table.** Comparison of LMEMs With City as Random Effect for Obesity Prevalence

LMEM model	AIC	BIC	*R* ^2^		Log likelihood	Deviance	Test	χ^2^	*P* value
			Marginal	Conditional					
SI	23 961	24 432	0.304	0.595	−11 906	23 813	NA	NA	NA
SV	23 099	23 735	0.413	0.663	−11 449	22 899	SI vs SV	914.05	<.001
SI + SV	22 650	23 737	0.525	0.717	−11 154	22 308	SV vs SI + SV	591.12	<.001
DSE	18 338	18 395	0.572	0.901	−9160	18 320			
DSE + SI + SV	17472	18 604	0.711	0.917	−8558	17 116	DSE vs DSE + SI + SV	1204	<.001
SDOH	21 173	21 212	0.552	0.775	−10 580.7	21 161			
SDOH + SI + SV	20 122	21 229	0.725	0.844	−9887.1	19 774	SDOH vs SDOH + SI + SV	1387.3	<.001
DSE + SDOH	17 949	18 025	0.632	0.905	−8962.5	17 925	NA	NA	NA
DSE + SDOH + SI + SV	16 984	18 135	0.745	0.926	−8310.8	16 622	DSE + SDOH vs DSE + SDOH + SI + SV	1303.4	<.001

Abbreviations: AIC, Akaike information criterion; BIC, bayesian information; DSE, demographic and socioeconomic factors (male sex, Black race, median age, Hispanic ethnicity, education level <high school, and median income); LMEM, linear mixed-effects model; NA, not applicable; SDOH, social determinants of health (social vulnerability index, socioeconomic disadvantage index, and area deprivation index); SI, satellite imagery–derived environmental features; SV, street view–derived environmental features.

The SI-only model had a marginal *R*^2^ of 0.304 and a conditional *R*^2^ of 0.595. Using SV features (SV-only model) had better performance in both metrics, with values of 0.413 and 0.663, respectively. The SV-only model (AIC = 23 099; BIC = 23 735) outperformed the SI-only model (AIC = 23 961; BIC = 24 432) based on both AIC and BIC. Combining both geospatial image features (SI + SV model) was associated with further improvement of the marginal *R*^2^ to 0.525 and conditional *R*^2^ to 0.717 (χ^2^ = 591.12; *P* < .001).

Models that included traditional risk factors outperformed those relying solely on geospatial imaging features. The DSE-only model achieved a marginal *R*^2^ of 0.572 and a conditional *R*^2^ of 0.901, demonstrating strong explanatory power. Adding geospatial imaging features to DSE (DSE + SI + SV model) was associated with improved model fit (χ^2^ = 1204; *P* < .001), with marginal and conditional *R*^2^ values increasing to 0.711 and 0.917, respectively. Similarly, the SDOH-only model had a marginal *R*^2^ of 0.552 and a conditional *R*^2^ of 0.775. Including SI and SV features (SDOH + SI + SV model) was associated with substantial improvements, with marginal *R*^2^ increasing to 0.725 and conditional *R*^2^ increasing to 0.844 (χ^2^ = 1387.3; *P* < .001).

**Figure 2.**
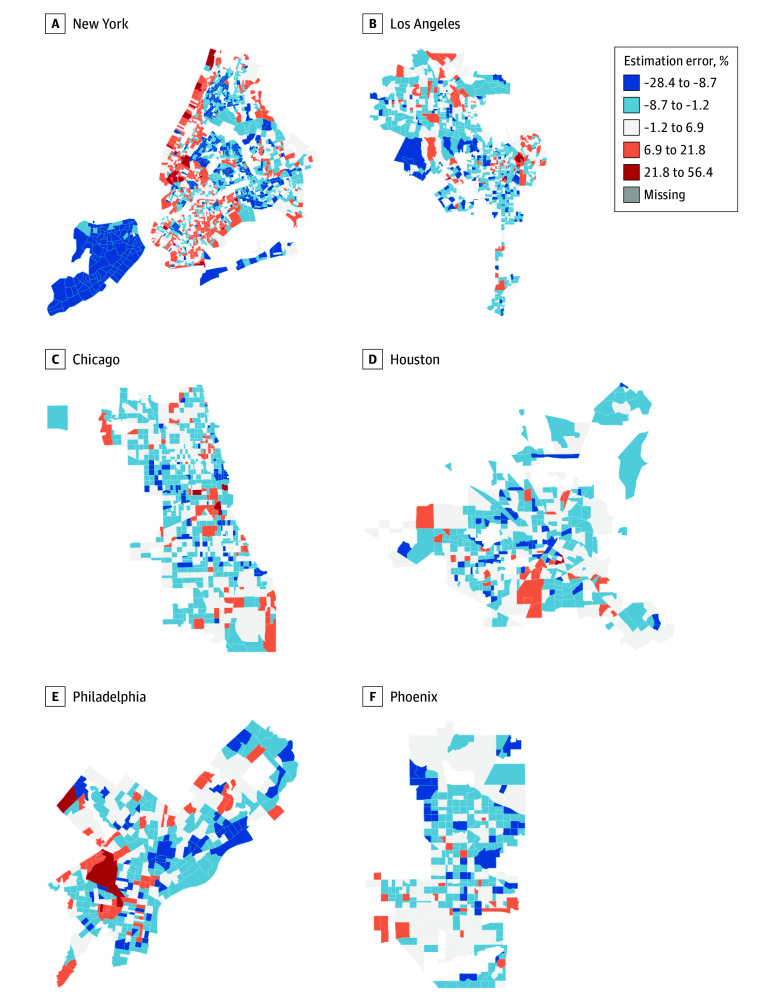
Estimation Error Maps for Top 6 US Cities Blue areas indicate underestimation, and red areas indicate overestimation by the model. Spatially clustered errors greater than approximately 10% may signal the presence of unique local health determinants (eg, targeted interventions or specific cultural dietary patterns) not captured by the model, flagging these communities for more focused, qualitative public health investigation.

The DSE plus SDOH model alone achieved a high marginal *R*^2^ of 0.632 and a conditional *R*^2^ of 0.905. Incorporation of geospatial imaging features (DSE + SDOH + SI + SV model) was associated with improved fit, with marginal and conditional *R*^2^ values of 0.745 and 0.926, respectively (eFigure 7 in Supplement 1). This model exhibited the best overall performance, with a χ^2^ value of 1303.4 (*P* < .001) compared with the model without geospatial imagery features (DSE plus SDOH model). Geographically, the model showed strong performance across cities (eFigure 8 in Supplement 1), with relative error rates mostly below 10%, although some areas showed overestimation or underestimation (Figure 2).

We performed 2 sensitivity analyses to confirm the robustness of our findings. First, when rerunning the models using older 2021 CDC PLACES data, the addition of geospatial features was still associated with improvement in estimating power (eTable 2 in Supplement 1). Second, a model that excluded the highly collinear social deprivation index and social vulnerability index yielded similar results (eTable 3 in Supplement 1), confirming that the incremental value of the image features was not dependent on a specific combination of SDOH factors (eTable 4 in Supplement 1).

### Visualization of Important Features With Grad-CAM

**Figure 3.**
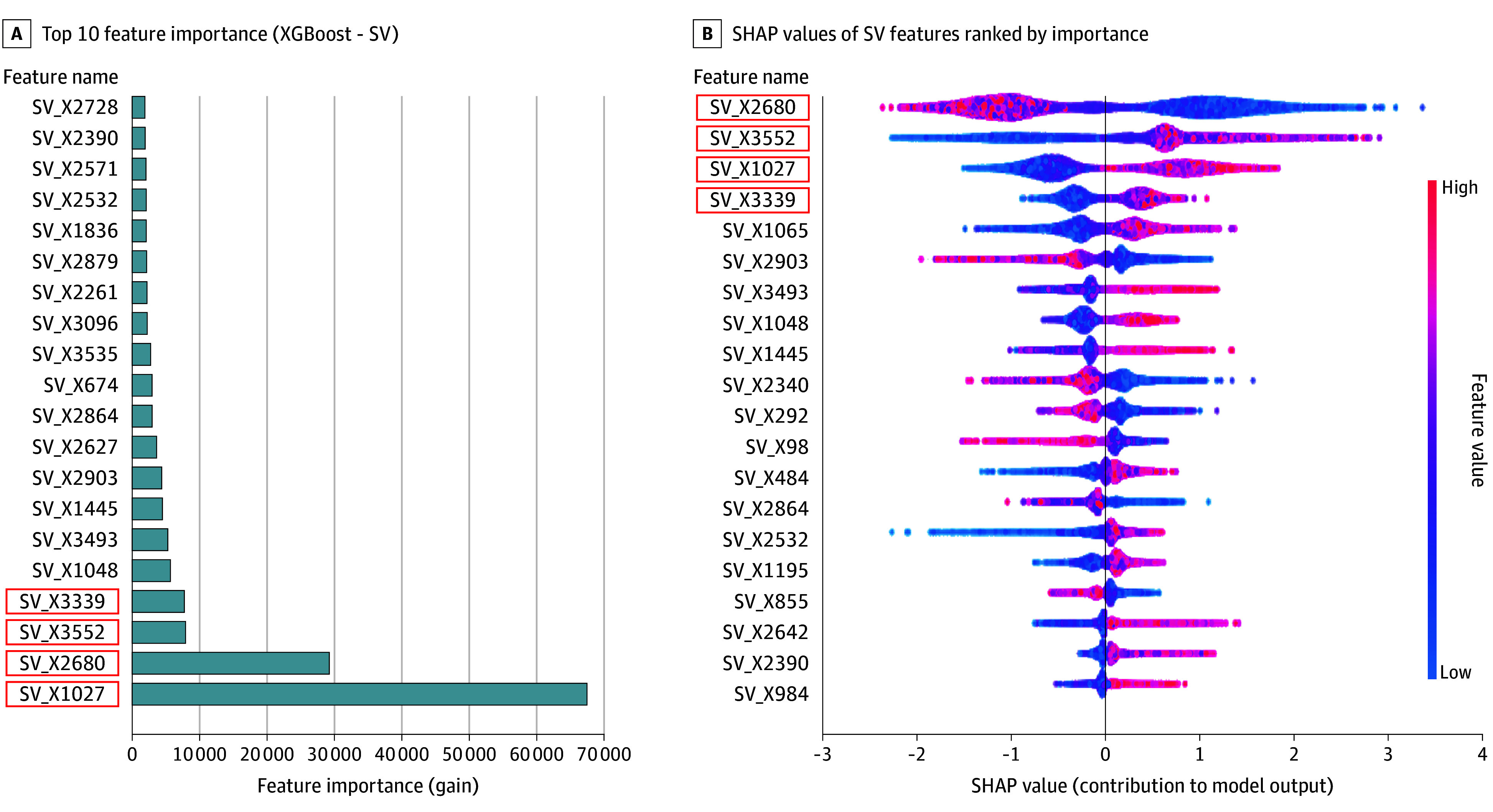
Importance and Interpretability of Street View (SV) Images in Association With Obesity Prevalence The figure deconstructs the artificial intelligence model to reveal which specific, built environment features from SV images were most important for estimating obesity. A, Feature importance of SV-derived features is based on the total gain in an XGBoost model. B, Shapley additive explanations (SHAP) values of SV features are ranked by importance. Positive SHAP values with low feature values (blue) indicate a negative association with obesity prevalence, while positive SHAP values with high feature values (red) indicate a positive association. Features highlighted in red boxes were consistently ranked among the most important by both methods.

**Figure 4.**
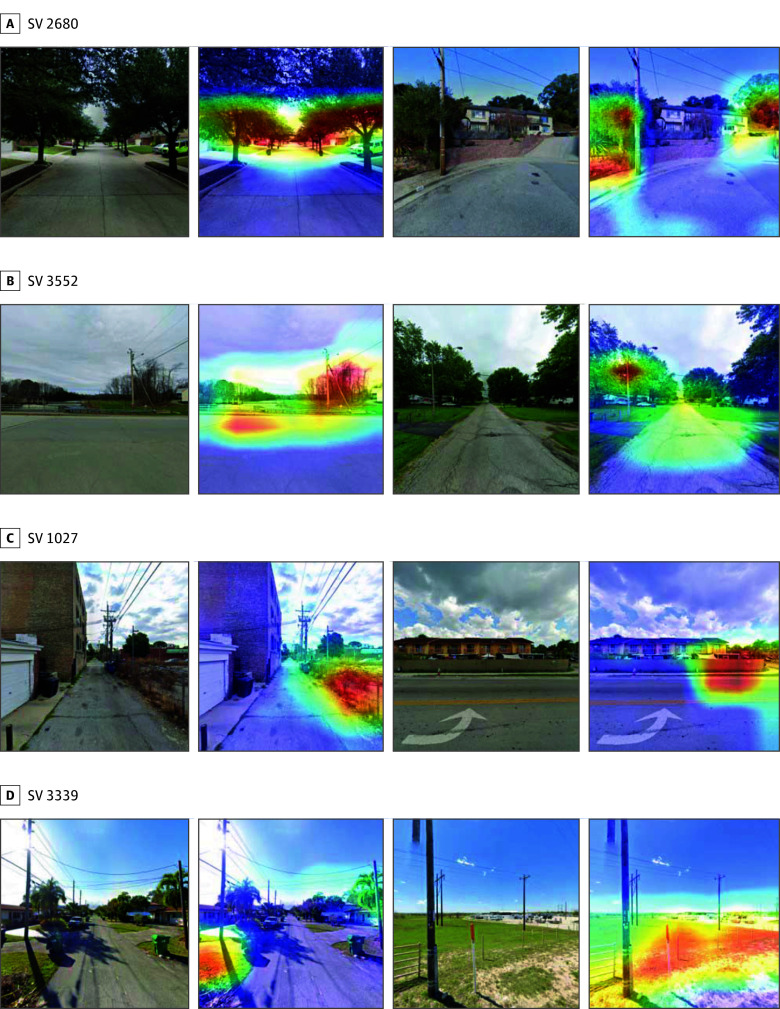
Visualizations of Key Street View (SV) Features This figure displays gradient-weighted class activation mapping heat maps for the top-ranked street view features, identified by feature importance and Shapley additive explanations values. Each pair shows the original image (left) and a heat map (right) highlighting regions most influential in feature extraction by the convolutional neural network. These regions predominantly capture built environment elements like trees (SV 2680), grass (SV 3339), fences (SV 1027), and utility poles (SV 3552).

As expected, several key features were consistently identified by feature importance (total gain) and SHAP values. Notably, SV 2680, SV 1027, SV 3552, and SV 3339 emerged as important SV-derived features (Figure 3A-B). Grad-CAM visualizations further revealed that SV 2680 primarily captured tree coverage, SV 1027 focused on fences, SV 3552 highlighted utility poles, and SV 3339 emphasized grassy areas (Figure 4). Among these features, SV 2680 (tree coverage) was associated with lower obesity prevalence, while SV 1027, SV 3552, and SV 3339 were associated with higher obesity prevalence, as indicated by the SHAP analysis. Key SI features included factories, road intersections, recreational fields, and highways, with recreational fields associated with lower obesity prevalence (eFigure 9 in Supplement 1). More grad-CAM SI and SV visualizations are provided in eFigures 10 and 11 in Supplement 1.

## Discussion

This cross-sectional study found that AI-analyzed geospatial imagery (SI and SV) provided significant explanatory power for census tract–level heterogeneity in US obesity prevalence, associated with substantial improvement in the performance of models that used traditional risk factors. Machine learning and mixed-effects models showed improved performance when SI and SV features were integrated, and further notable enhancement occurred when these were combined with conventional DES and SDOH risk factors. This highlights the incremental benefit associated with geospatial data, demonstrating the potential for integrated approaches in creating robust population health strategies that consider spatial environments associated with obesity risk.

A novel contribution of our study is the integration of advanced deep learning techniques analyzing multimodal geospatial imagery (SI and SV imagery) with traditional DSE and SDOH variables within a hierarchical mixed-effects modeling framework. This approach advances the field by capturing macro- and micro-level environmental details. Unlike many previous studies that used single imaging modalities, manual feature extraction, or regression models that do not account for geographic clustering,^32,33^ our use of mixed-effects models specifically addressed intracity variability and area-level clustering effects, thereby isolating the contribution of fixed effects and offering more robust and precise estimates of the built environment’s association with obesity.

The superior performance of SV features likely stems from their ability to capture high-resolution, street-level details that are more closely aligned with daily human experiences of the built microenvironment. These images reveal nuanced factors, such as street trees, pedestrian infrastructure, storefronts, and other microscale elements that may be associated with physical activity and, consequently, obesity risk.^15^ This richness in visual detail likely also explains why our SPLS model required more components to summarize SV data (*K* = 97) compared with the more-uniform satellite data (*K* = 71). However, SI images offer a broader, more generalized view and provide global coverage, especially when SV may be unavailable. The combined model’s enhanced performance further underscores the complementary nature of these 2 data modalities. While SV features deliver detailed, street-level insights, SI features contribute macrolevel context regarding overall land use and environmental patterns. Integrating both allowed for a more comprehensive representation of the built environment, leading to improved estimation of obesity prevalence. This synergy not only boosted model accuracy but also reinforces the potential of leveraging multiscale geospatial imaging data to uncover critical environmental determinants of public health outcomes.

A key strength of the integrated model was the substantial improvement in LMEM fit when geospatial imaging features were incorporated with traditional DSE and SDOH factors. The marginal *R*^2^ increased from 0.632 to 0.745, suggesting that image-derived features provide an independent contribution to explaining neighborhood-level obesity prevalence and capturing intracity variance. The conditional *R*^2^ also improved, showing that features may have captured additional variance between cities as well. This improvement was not merely due to model complexity, as confirmed by significantly lower AIC and BIC and significant likelihood ratio tests. Collectively, these metrics suggest that integrating geospatial data offers a robust and parsimonious enhancement to conventional obesity models.

The geographic variation in model performance is also informative. For instance, areas with consistent underestimation of obesity could suggest the presence of unmeasured risk factors, like food environment,^45^ while overestimation could point to protective factors, such as social cohesion and social capital.^46^ These variations underscore that while our model is powerful, it is best used as a tool to highlight areas where deeper, context-specific investigation is warranted.

Earlier studies have shown that predefined features, such as walkability, green space, and access to recreational facilities, are associated with lower obesity prevalence and improved physical activity levels.^16,47^ Our grad-CAM visualizations offered interpretable, image-derived insights into how specific built environment features were associated with obesity, largely reinforcing known associations. For instance, the negative association of tree coverage with obesity prevalence aligns with recognized green space benefits,^48,49^ while positive associations for features like fences and utility poles may indicate less activity-promoting environments, potentially characteristic of suburban sprawl.^50^ While road intersections (SI) showed a positive association with obesity,^51^ the broader SI findings of highways and factories being associated with higher obesity prevalence could reflect negative outcomes of traffic, pollution, and reduced walkability near major roads and industrial zones.^52^ Recreational fields (SI) were, as expected, associated with lower obesity prevalence, reinforcing the importance of accessible recreation.^53^ Overall, grad-CAM results reinforced known associations between built environment features and obesity prevalence, offering image-derived insights into these complex associations.

Our findings have significant implications for public health and urban planning. The enhanced performance associated with integrating geospatial imaging with traditional data underscores the crucial role of built environment factors, particularly street-level details. This suggests that policies promoting urban designs favoring walkability, green spaces, and active transportation are vital for potential reductions in obesity prevalence. For example, improved pedestrian infrastructure and mixed land-use planning are associated with better health outcomes.^54,55^ This work also has clinical implications. Clinicians may leverage high-resolution risk maps derived from this freely available imagery to inform counseling on environment-related behaviors, like physical activity. This allows for a deeper understanding of patients’ environmental context.

### Limitations

These findings should be interpreted within the context of study limitations. Although a sensitivity analysis confirmed our findings’ robustness, the temporal mismatch between the primary imaging and outcome data constitute a limitation that may have introduced misclassification bias. Additionally, the reliance on self-reported data for certain variables, along with varying frequencies in image updates, could introduce biases that may influence overall findings. The generalizability of our results is also somewhat constrained by the specific selection of cities and census tracts included in the study, which may not fully represent other geographic regions or urban contexts. Furthermore, model-based estimates derived from sources, such as the BRFSS and ACS, that inherently carry limitations that could affect the precision of our obesity prevalence measures. Our analysis does not account for the recent, increasing use of glucagon-like peptide-1 receptor agonist medications for weight management, but this use may have started to influence neighborhood obesity rates. Moreover, the interpretation of the deep learning features relied on a qualitative visual assessment, which awaits a more systematic, multirater validation. Additionally, our approach may need to be tested in rural environments, which have substantially different geospatial features.

## Conclusions

In this cross-sectional study, we demonstrated that integrating deep learning–derived geospatial features from SI and SV imagery was associated with improved estimation of neighborhood obesity prevalence, especially when combined with traditional DSE and SDOH factors. This multimodal approach offered a more comprehensive understanding of built environment associations, highlighting the potential of geospatial imagery to inform targeted public health interventions and urban planning strategies.

## Notes

Supplement 1.
eMethods.
**eFigure 1.** Distribution of the 93 largest cities in the US, selected for study based on population size **eFigure 2.** Framework for obesity prevalence modeling using AI-enhanced image preprocessing (satellite and street view images) and statistical analysis, integrating deep learning features, sociodemographic covariates, and social determinants of health indices **eFigure 3.** Obesity crude prevalence across 5 cities with the highest and lowest obesity prevalence, with census tract counts (N) indicated for each city **eFigure 4.** Cross-validation mean squared estimation error (CV MSPE) plots for SPLS training results **eFigure 5.** UMAP projection of GSI **eFigure 6.** UMAP projection of GSV **eFigure 7.** Estimated vs observed obesity prevalence **eFigure 8.** Comparison of observed crude obesity prevalence (left), derived from CDC 2023 PLACES dataset and model-estimated crude obesity prevalence (right) across top 6 US cities: New York, Los Angeles, Chicago, Houston, Philadelphia, and Phoenix **eFigure 9.** Importance and interpretability of satellite image (SI) features in association with obesity prevalence. **eFigure 10.** Additional grad-CAM visualization of SI features **eFigure 11.** Additional grad-CAM visualization of SV features **eTable 1.** Basic characteristics of census tracts by quartile of obesity prevalence **eTable 2.** Sensitivity analysis of model performance for obesity prevalence using 2021 CDC PLACES data **eTable 3.** Variance inflation factor (VIF) values for key demographic, socioeconomic, and SDOH factors **eTable 4.** Sensitivity analysis of model performance for obesity prevalence after removing SDI and SVI

Supplement 2.
Data Sharing Statement
