# Error in Author Name

**DOI:** 10.1001/jamanetworkopen.2025.54305

**Published:** 2025-12-23

In the Original Investigation titled “AI-Enhanced Analysis of Built Environment Imagery and Neighborhood Obesity in US Cities,”^1^ published September 30, 2025, author Sai Rahul Ponnana’s name was misspelled. The article has been corrected.^1^

## References

[1] Chen Z, Zhang T, Dazard JE, . AI-enhanced analysis of built environment imagery and neighborhood obesity in US cities. JAMA Netw Open. 2025;8(9):e2534612. doi:10.1001/jamanetworkopen.2025.34612 41026491 PMC12485642

