# Supplementary material for: AI-Enhanced Analysis of Built Environment Imagery and Neighborhood Obesity in US Cities
Source: JAMA Netw Open. 2025 Sep 30;8(9):e2534612. doi: 10.1001/jamanetworkopen.2025.34612 (PMC12485642; doi:10.1001/jamanetworkopen.2025.34612)
Supplement: Supplement 1. — eMethods. eFigure 1. Distribution of the 93 largest cities in the US, selected for study based on population size eFigure 2. Framework for obesity prevalence modeling using AI-enhanced image preprocessing (satellite and street view images) and statistical analysis, integrating deep learning features, sociodemographic covariates, and social determinants of health indices eFigure 3. Obesity crude prevalence across 5 cities with the highest and lowest obesity prevalence, with census tract counts (N) indicated for each city eFigure 4. Cross-validation mean squared estimation error (CV MSPE) plots for SPLS training results eFigure 5. UMAP projection of GSI eFigure 6. UMAP projection of GSV eFigure 7. Estimated vs observed obesity prevalence eFigure 8. Comparison of observed crude obesity prevalence (left), derived from CDC 2023 PLACES dataset and model-estimated crude obesity prevalence (right) across top 6 US cities: New York, Los Angeles, Chicago, Houston, Philadelphia, and Phoenix eFigure 9. Importance and interpretability of satellite image (SI) features in association with obesity prevalence. eFigure 10. Additional grad-CAM visualization of SI features eFigure 11. Additional grad-CAM visualization of SV features eTable 1. Basic characteristics of census tracts by quartile of obesity prevalence eTable 2. Sensitivity analysis of model performance for obesity prevalence using 2021 CDC PLACES data eTable 3. Variance inflation factor (VIF) values for key demographic, socioeconomic, and SDOH factors eTable 4. Sensitivity analysis of model performance for obesity prevalence after removing SDI and SVI [file jamanetwopen-e2534612-s001.pdf]

Supplemental Online Content

Chen Z, Zhang T, Dazard JE, et al. AI-enhanced analysis of built environment imagery and neighborhood obesity in US cities. *JAMA Netw Open*. 2025;8(9):e2534612.
doi:10.1001/jamanetworkopen.2025.34612

eMethods.

eFigure 1. Distribution of the 93 largest cities in the US, selected for study based on population size

eFigure 2. Framework for obesity prevalence modeling using AI-enhanced image preprocessing (satellite and street view images) and statistical analysis, integrating deep learning features, sociodemographic covariates, and social determinants of health indices

eFigure 3. Obesity crude prevalence across 5 cities with the highest and lowest obesity prevalence, with census tract counts (N) indicated for each city

eFigure 4. Cross-validation mean squared estimation error (CV MSPE) plots for SPLS training results

eFigure 5. UMAP projection of GSI

eFigure 6. UMAP projection of GSV

eFigure 7. Estimated vs observed obesity prevalence

eFigure 8. Comparison of observed crude obesity prevalence (left), derived from CDC 2023 PLACES dataset and model-estimated crude obesity prevalence (right) across top 6 US cities: New York, Los Angeles, Chicago, Houston, Philadelphia, and Phoenix

eFigure 9. Importance and interpretability of satellite image (SI) features in association with obesity prevalence.

eFigure 10. Additional grad-CAM visualization of SI features

eFigure 11. Additional grad-CAM visualization of SV features

eTable 1. Basic characteristics of census tracts by quartile of obesity prevalence

eTable 2. Sensitivity analysis of model performance for obesity prevalence using 2021 CDC PLACES data

eTable 3. Variance inflation factor (VIF) values for key demographic, socioeconomic, and SDOH factors

eTable 4. Sensitivity analysis of model performance for obesity prevalence after removing SDI and SVI

This supplemental material has been provided by the authors to give readers additional information about their work.

© 2025 Chen Z et al. *JAMA Network Open*.

eMethods

Image Data Collection and Feature Extraction

For GSI, we sampled up to five points per census block group, ensuring a minimum 500-meter separation, with triangulation employed for even spatial distribution. GSI images were retrieved at zoom level 16, providing an approximate spatial resolution of 2.4 meters. For GSV, five points were randomly sampled per block group, maintaining a minimum 100-meter separation between points along the road network, with images captured in the North, West, South, and East directions. Images were retrieved based on the sampled points from May to July 2024 using Google Maps API. It's important to note the temporal variability inherent in Google image datasets. Google Satellite Images are often cloud-free mosaics compiled from imagery captured at different times, mainly reflecting the ground environment from 2022-2023 in our dataset. Temporal variation is even more pronounced in GSV, with image dates ranging from as early as 2010 in some rural areas to as recent as the image collection period (2024).

We employed a pretrained convolutional neural network (ResNet-50)[1] to extract deep features from both GSI and GSV images. For the GSI images, we began with a ResNet-50 model pretrained on ImageNet and fine-tuned it using the UC Merced Land Use dataset[2]. We then applied this fine-tuned model to the GSI images and extracted 4,096 features from the fully connected layers. This process converts the visual information within the images into a numerical feature vector. These extracted features served as input variables for our subsequent obesity prevalence prediction model. A similar procedure was followed for the GSV images, except that we used a ResNet-50 model pretrained on the 365 Places dataset[3]. Places365 consists of millions of images, providing a substantial amount of data with a wide variety of scene categories, which helps models learn to recognize a diverse range of environments. For each census tract, we aggregated the 4,096 features extracted from all GSI or GSV images by calculating the mean for each feature. This resulted in a 4,096-dimensional 'built environment signature' representing each tract[2,4]. Our primary rationale for choosing the mean for aggregation was to generate a single, holistic 'built environment signature' for each census tract, which is a standard and robust approach for this type of area-level analysis. In our preliminary experiments, we also compared different aggregation methods (e.g., mean vs. max) and found that using the mean consistently resulted in features with stronger predictive performance in explaining the variation in obesity prevalence.

Sparse Partial Least Squares (SPLS) for Feature Reduction

Given the high dimensionality of the GSI and GSV feature sets (p=4,096), **Sparse Partial Least Squares (SPLS) regression** was employed for simultaneous dimensionality reduction and variable selection. SPLS identifies a set of latent components that explain covariance between the predictors and the outcome, while also inducing sparsity by forcing some predictor coefficients to zero. This process was performed independently on the GSI and GSV training sets.

A 10-fold **cross-validation** procedure was used to tune the model's two key hyperparameters:

1. **K**: The number of hidden components to extract.
2. **η (eta)**: The sparsity parameter (ranging from 0.1 to 0.9), which controls the number of predictors selected for each component.

The optimal combination of K and η was selected by identifying the pair that minimized the cross-validated mean squared prediction error (CV MSPE).

- For **GSI features**, the optimal hyperparameters were determined to be K=71 and η=0.5.
- For **GSV features**, the optimal hyperparameters were K=97 and η=0.4.

The cross-validation performance for this tuning process is visualized in **eFigure 3**.

Generation of Hidden Components for the Test Set

Using the optimal hyperparameters identified above, final SPLS models were fitted to the entire training dataset for both GSI and GSV. To generate the corresponding hidden components for the independent test set, a custom function was implemented that incorporates a correction procedure to enhance the robustness of the feature selection.

This procedure involved the following steps:

1. **Bootstrap Confidence Intervals**: For the fitted SPLS model, 1,000 bootstrap samples were run to calculate 95% confidence intervals for the model coefficients.
2. **Coefficient Correction**: A correction was applied to the SPLS coefficients. Only predictors whose bootstrap confidence intervals did not contain zero were retained, ensuring that the selected features had a stable, non-zero relationship with the outcome.

3. **Component Projection**: The trained projection matrix from the corrected SPLS model was then applied to the corresponding test set data to generate the final hidden components.

This process resulted in a set of 71 hidden components for GSI and 97 for GSV for each census tract in the test set. These newly generated components, representing the key features of the built environment, were then merged with the other demographic and socioeconomic variables in the test set for use as fixed effects in the final Linear Mixed-Effects Model (LMEM) analysis.

Machine Learning Model Development and Interpretation

We developed an XGBoost regression model to predict obesity crude prevalence using GSI and/or GSV features extracted from convolutional neural networks. The dataset was split into training (70%) and testing (30%) sets using stratified sampling based on the cities.

Hyperparameter optimization was performed using Optuna[5] in conjunction with 5-fold cross-validation. The objective function optimized the mean coefficient of determination (R^2) score on the training folds. We constrained the search space to the following ranges:

- Lambda: 1e-3 to 10.0 (log scale)
- Alpha: 1e-3 to 10.0 (log scale)
- Learning rate: 0.01 to 0.3 (log scale)
- Number of estimators: 75 to 125
- Max depth: 3 to 15

After completing five optimization trials using Optuna, the best hyperparameters for the XGBoost model were identified and subsequently used for final training. The model, designed to predict obesity crude prevalence, was evaluated on independent test data using the R^2 and mean squared error (MSE), with the MSE additionally reported as root mean squared error (RMSE). Experiments were conducted on three datasets: one incorporating GSV features only, another with GSI features only, and a third combining both GSV and GSI features. For interpretability, SHAP (SHapley Additive exPlanations) was utilized to quantify the contributions of individual features. SHAP values were calculated via a TreeExplainer customized for the final XGBoost model.

Machine learning model performance

The machine learning models were evaluated on the test set. The combined GSV+GSI model showed the strongest predictive performance, with an R^2 of 0.70, an MSE of 21.21, and an RMSE of 4.61. This model significantly outperformed both the GSV-only model (R^2: 0.66, MSE: 23.89, RMSE: 4.89) and the GSI-only model (R^2: 0.59, MSE: 28.96, RMSE: 5.38).

Supplemental References:

1. He K, Zhang X, Ren S, Sun J. Deep residual learning for image recognition. In: *Proceedings of the IEEE Computer Society Conference on Computer Vision and Pattern Recognition*. IEEE Computer Society; 2016:770-778. doi:10.1109/CVPR.2016.90

2. Chen Z, Dazard JE, Khalifa Y, et al. Deep Learning–Based Assessment of Built Environment From Satellite Images and Cardiometabolic Disease Prevalence. *JAMA Cardiol*. Published online May 1, 2024. doi:10.1001/jamacardio.2024.0749

3. Zhou B, Lapedriza A, Khosla A, Oliva A, Torralba A. Places: A 10 Million Image Database for Scene Recognition. *IEEE Transactions on Pattern Analysis and Machine Intelligence*. 2018;40(6):1452-1464. doi:10.1109/TPAMI.2017.2723009

4. Chen Z, Dazard JE, Khalifa Y, Motairek I, Al-Kindi S, Rajagopalan S. Artificial intelligence–based assessment of built environment from Google Street View and coronary artery disease prevalence. *European Heart Journal*. Published online March 28, 2024:ehae158. doi:10.1093/eurheartj/ehae158

5. Akiba T, Sano S, Yanase T, Ohta T, Koyama M. Optuna: A Next-generation Hyperparameter Optimization Framework. In: *Proceedings of the 25th ACM SIGKDD International Conference on Knowledge Discovery & Data Mining*. KDD ’19. Association for Computing Machinery; 2019:2623-2631. doi:10.1145/3292500.3330701

© 2025 Chen Z et al. *JAMA Network Open*.

Supplemental Figures

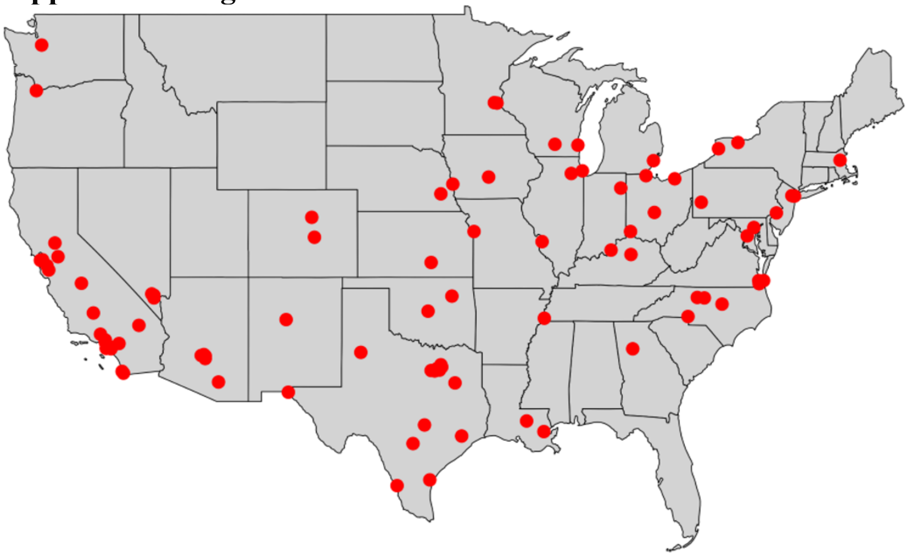

eFigure 1. Distribution of the 93 largest cities in the United States, selected for study based on population size. Florida cities are excluded from the 100 cities due to lack of data in 2023 CDC PLACES.

© 2025 Chen Z et al. *JAMA Network Open*.

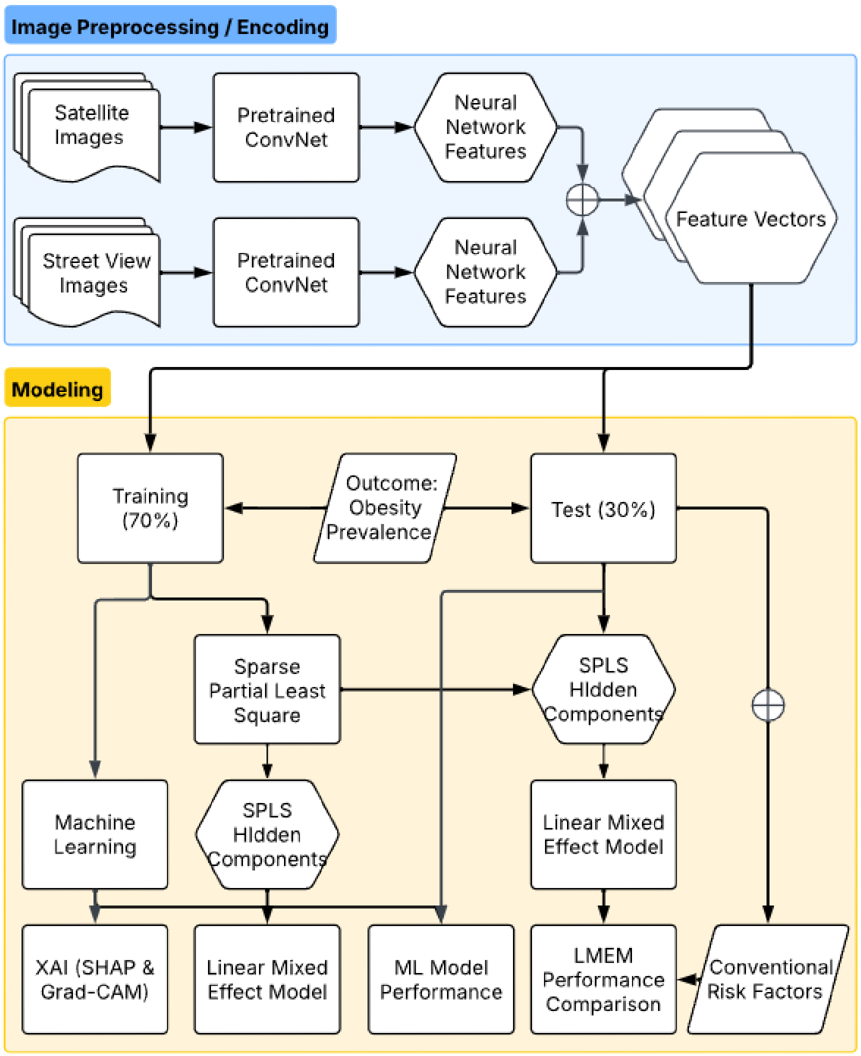

eFigure 2. Framework for obesity prevalence modeling using AI-enhanced image preprocessing (satellite and street view images) and statistical analysis, integrating deep learning features, socio-demographic covariates, and social determinants of health indices.

© 2025 Chen Z et al. *JAMA Network Open*.

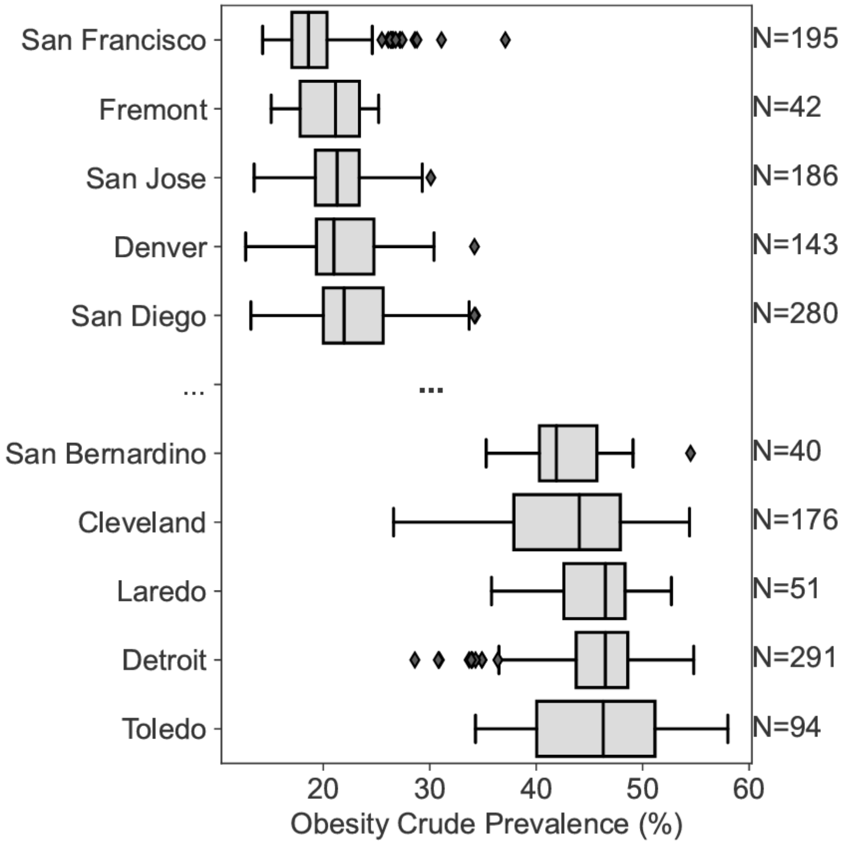

eFigure 3. Obesity crude prevalence across the 5 cities with the highest and lowest obesity prevalence, with census tract counts (N) indicated for each city.

© 2025 Chen Z et al. *JAMA Network Open*.

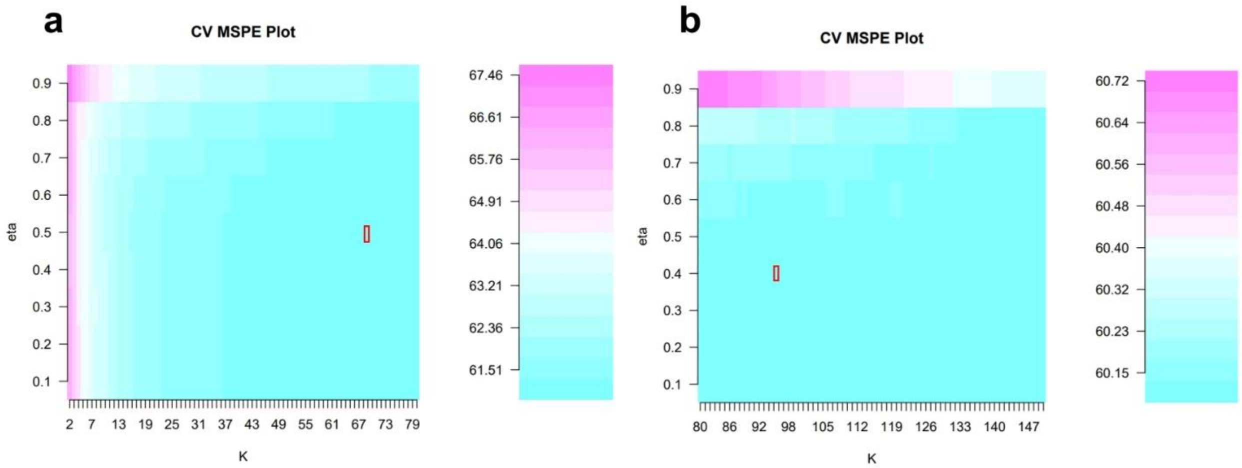

eFigure 4. Cross-validation mean squared estimation error (CV MSPE) plots for SPLS training results. **a)** GSI feature optimization: the optimal combination of η=0.5 and K=71 is highlighted in red, minimizing CV MSPE. **b)** GSV feature optimization: the optimal combination of η=0.4 and K=97 is highlighted in red, achieving the lowest CV MSPE. The heatmaps display CV MSPE across different values of η (sparsity parameter) and K (number of components). Overall, this process minimizes prediction error and prevents overfitting. The red boxes indicate the most accurate versions of the models, which were used for the final analysis.

© 2025 Chen Z et al. *JAMA Network Open*.

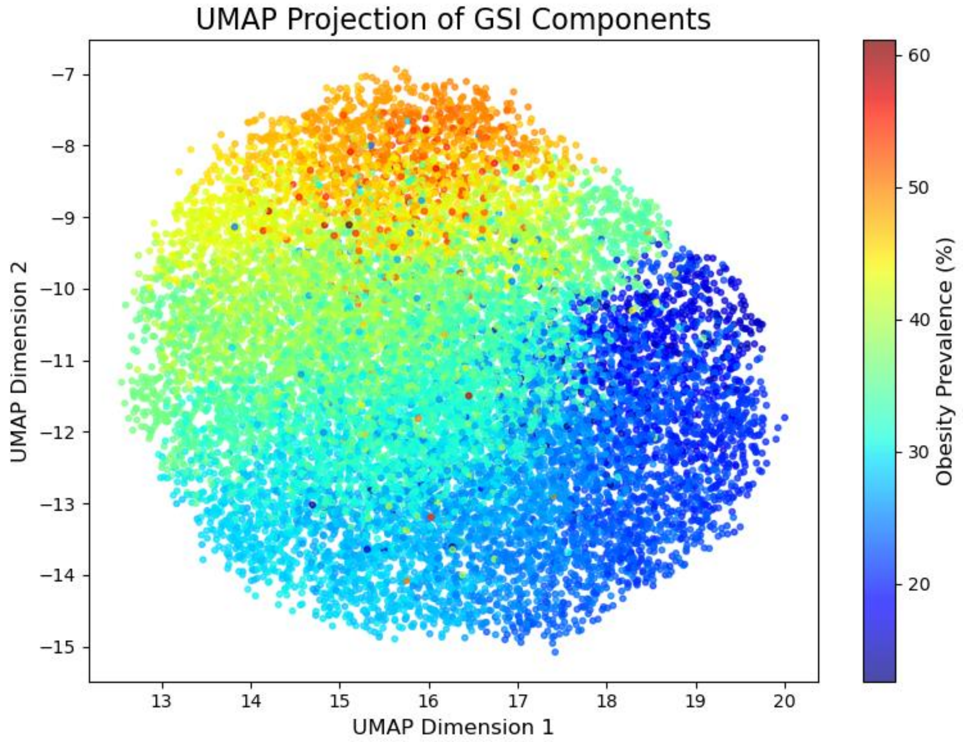

eFigure 5. UMAP Projection of GSI. UMAP is used to group census tracts based on similarities in GSI (satellite images). Each dot is a census tract, colored by its obesity rate. Notice the clear separation where neighborhoods with lower obesity (blue dots) cluster together, apart from neighborhoods with higher obesity (yellow and red dots). This visually confirms that features captured in satellite images are strongly associated with a community's obesity prevalence.

© 2025 Chen Z et al. *JAMA Network Open*.

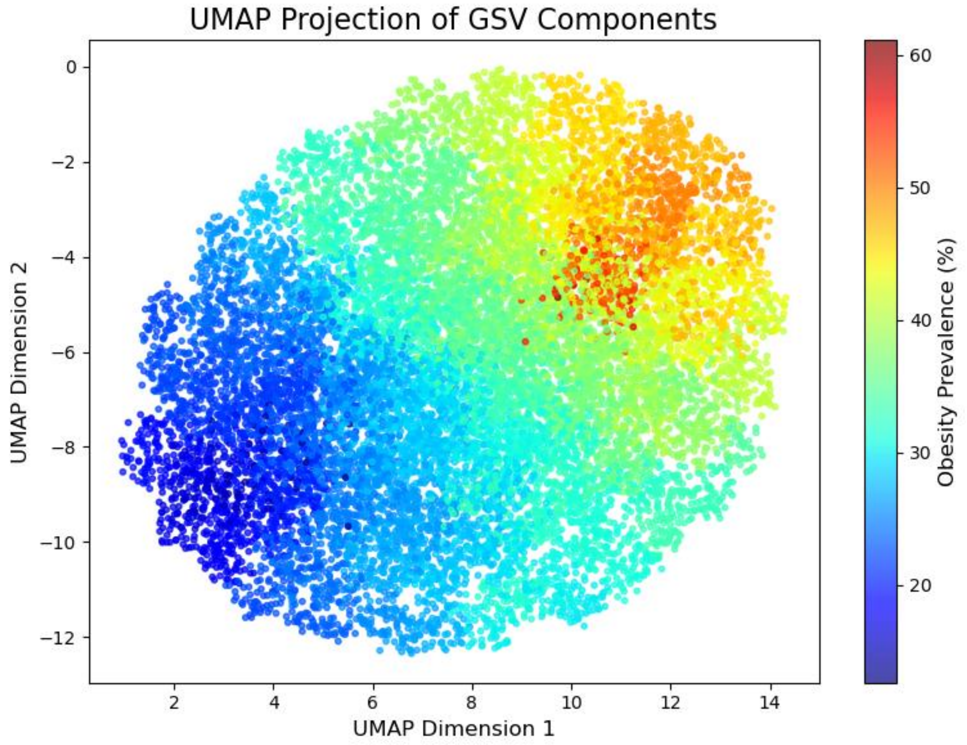

eFigure 6. UMAP Projection of GSV. UMAP is used to group census tracts based on similarities in GSV (street view images). Each dot is a census tract, colored by its obesity rate. Notice the clear separation where neighborhoods with lower obesity (blue dots) cluster together, apart from neighborhoods with higher obesity (yellow and red dots). This visually confirms that features captured in street view images are strongly associated with a community's obesity prevalence.

© 2025 Chen Z et al. *JAMA Network Open*.

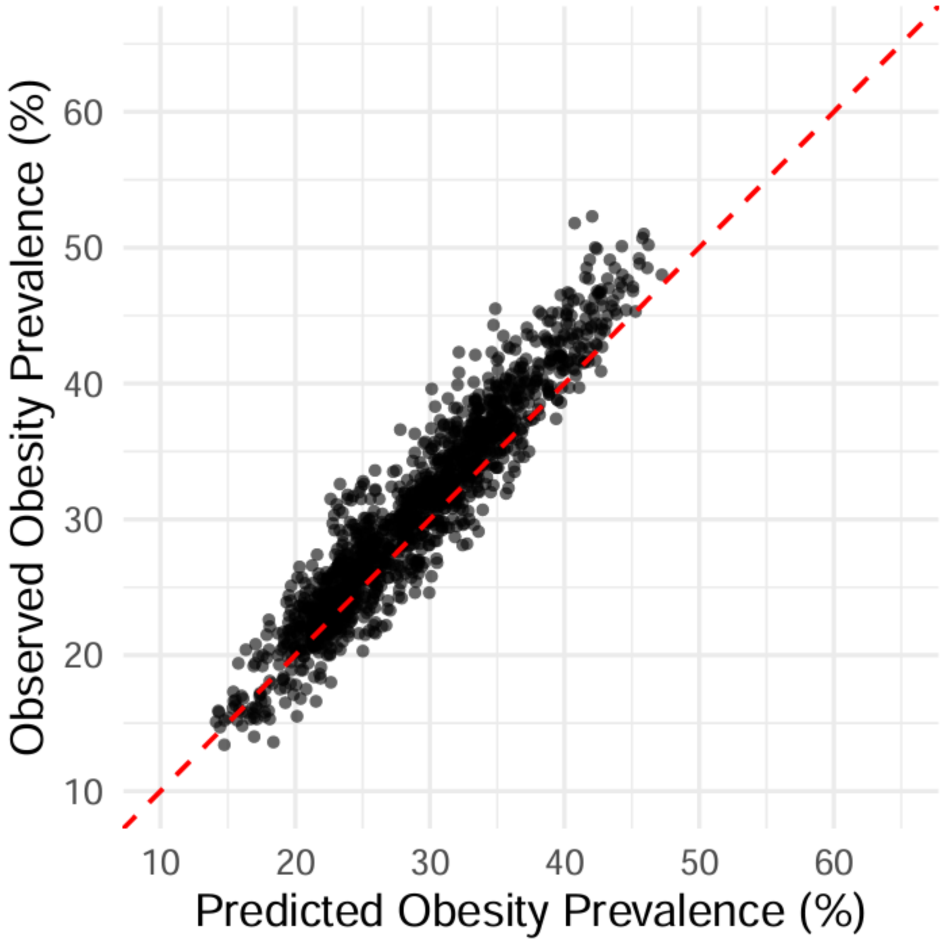

eFigure 7. Estimated vs. Observed Obesity Prevalence. The full model, integrating satellite and street view features with other risk factors, demonstrates high accuracy, explaining 92.6% of the variance in obesity prevalence within the hold-out test set (30%). The dashed red line represents perfect agreement between predicted and observed values.

© 2025 Chen Z et al. *JAMA Network Open*.

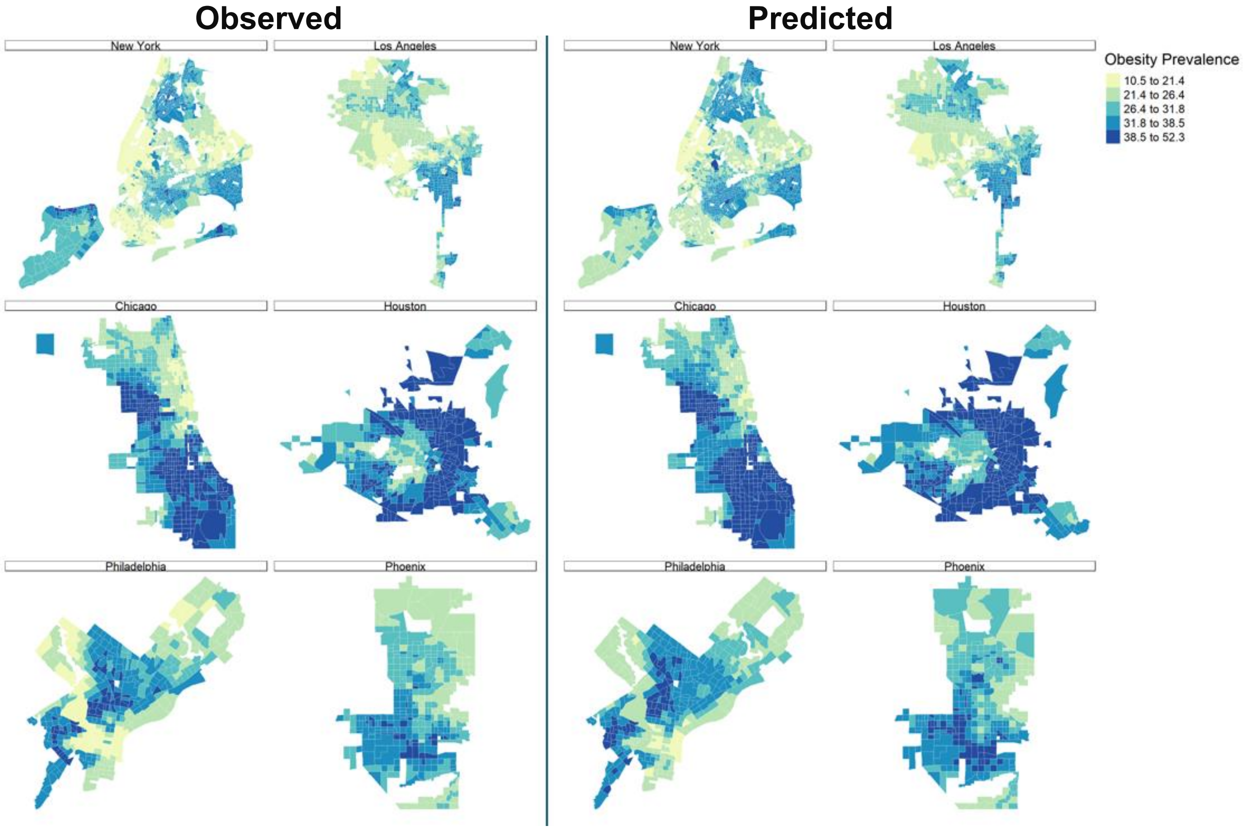

eFigure 8. Comparison of observed crude obesity prevalence (left), derived from CDC's 2023 PLACES dataset, and model-predicted crude obesity prevalence (right) across the top six U.S. cities: New York, Los Angeles, Chicago, Houston, Philadelphia, and Phoenix. The color scale is consistent across both sets of maps, with lighter shades indicating lower prevalence and darker shades representing higher prevalence. The strong visual correspondence between the observed and predicted patterns within each city demonstrates the model's high level of performance in capturing geographic variations in obesity rates.

© 2025 Chen Z et al. *JAMA Network Open*.

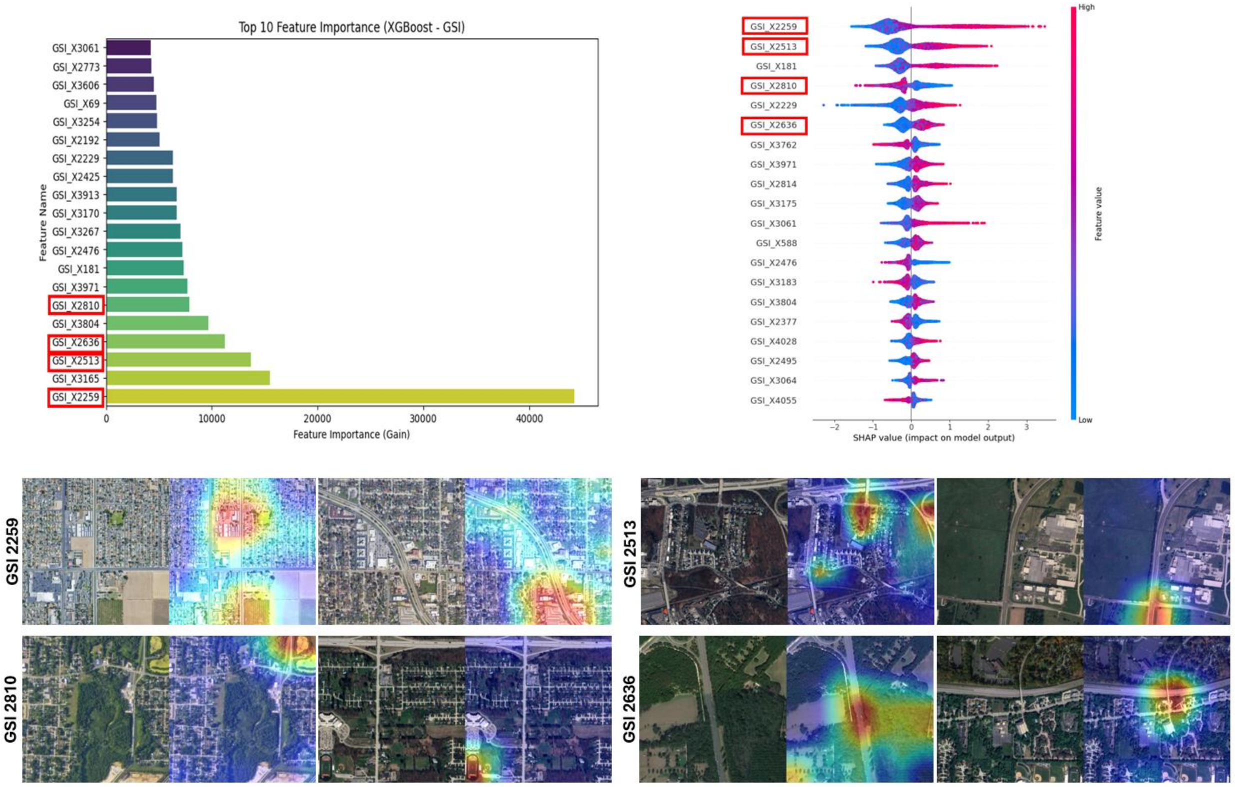

eFigure 9. Importance and interpretability of Satellite Image (GSI) features in relation to obesity prevalence. **a)** Feature importance of GSI-derived features based on the total gain in an XGBoost model. **b)** SHAP values of the GSV features, ranked by importance. Positive SHAP values with low feature values (blue) indicate a negative association with obesity prevalence, while positive SHAP values with high feature values (red) indicate a positive association. Features highlighted in red boxes are consistently ranked among the most important by both methods. **c)** Grad-CAM visualizations for the top-ranked GSI features, highlighting image regions most influential in feature extraction by the convolutional neural network (CNN). These regions predominantly capture built environmental elements such as factories (GSI 2259), road intersections (GSI 2513), recreation fields (GSI 2810), and highways (GSI 2636).

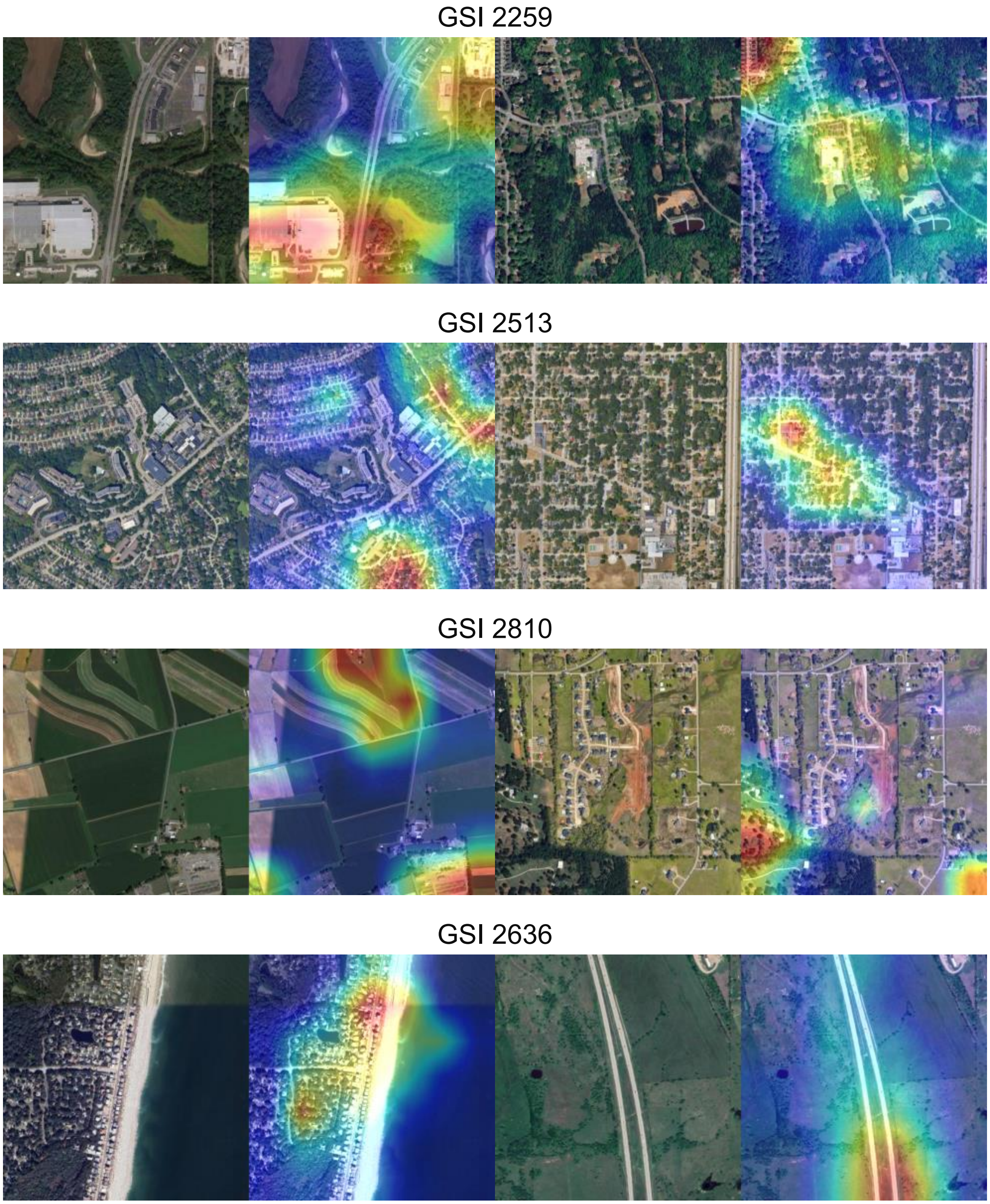

eFigure 10. **Additional Grad-CAM visualization of GSI features.** Each pair shows the original Google Satellite Image on the left and the corresponding Grad-CAM heatmap overlay on the right. "Hot" areas (red/yellow) indicate high importance, while "cool" areas (blue) are less important. With some noises, this provides more examples for the built environment features: factories (GSI 2259), road intersections (GSI 2513), recreation fields (GSI 2810), and highways (GSI 2636).

© 2025 Chen Z et al. *JAMA Network Open*.

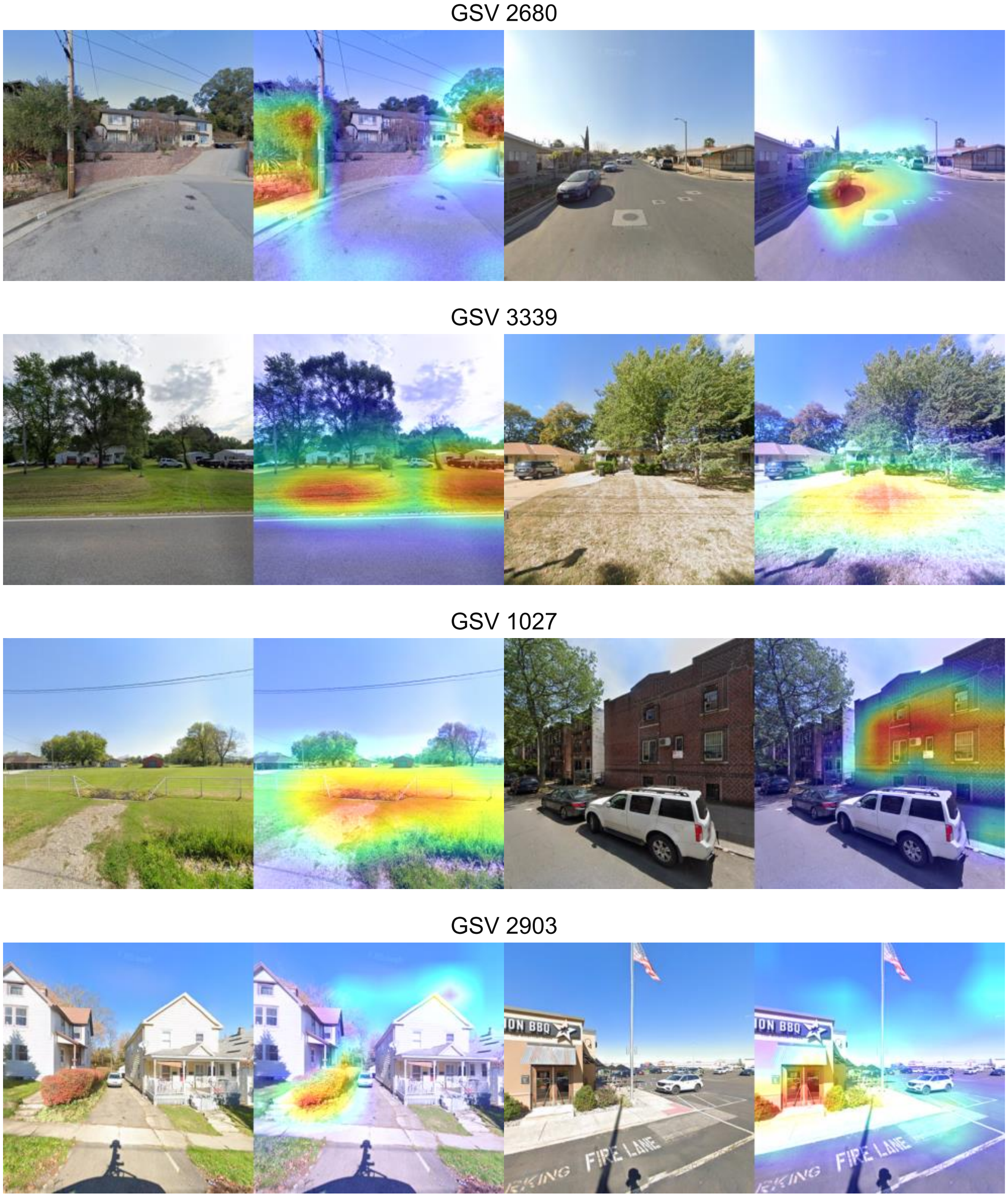

eFigure 10. Additional Grad-CAM visualization of GSV features. Each pair shows the original Google Satellite Image on the left and the corresponding Grad-CAM heatmap overlay on the right. "Hot" areas (red/yellow) indicate high importance, while "cool" areas (blue) are less important. With some noises, this provides more examples for the built environment features: trees (GSV 2680), grass (GSV 3339), fences (GSV 1027), and foundation shrubs/bushes (GSV 2903).

© 2025 Chen Z et al. *JAMA Network Open*.

Supplemental Tables

eTable 1. Basic Characteristics of the census tracts by quartiles of obesity prevalence.

Characteristic	**All,** N = 14,413[1]	**Q1 (Lowest)**, N = 3,604[1]	**Q2**, N = 3,603[1]	**Q3**, N = 3,603[1]	**Q4 (Highest)**, N = 3,603[1]
Obesity Prevalence (%)	32.4 (26.6, 38.8)	23.0 (20.6, 24.9)	29.6 (28.1, 31.1)	35.4 (33.9, 37.0)	43.6 (40.9, 46.9)
Total Population	3,861 (2,701, 5,210)	3,979 (2,917, 5,297)	4,018 (2,929, 5,315)	4,180 (2,936, 5,481)	3,200 (2,147, 4,590)
Social Deprivation Index (SDI)	0.71 (-0.19, 1.35)	-0.10 (-0.65, 0.53)	0.18 (-0.59, 0.89)	0.88 (0.21, 1.41)	1.44 (1.12, 1.67)
Social Vulnerability Index (SVI)	0.66 (0.33, 0.86)	0.33 (0.16, 0.58)	0.46 (0.21, 0.72)	0.73 (0.48, 0.89)	0.88 (0.78, 0.95)
Area Deprivation Index (ADI)	40 (16, 72)	10 (5, 19)	29 (16, 45)	53 (31, 69)	87 (73, 95)
Female Population (%)	51.1 (48.7, 53.8)	50.8 (48.4, 53.1)	50.9 (48.7, 53.1)	51.0 (48.5, 53.7)	52.0 (49.1, 55.2)
Male Population (%)	48.9 (46.2, 51.3)	49.2 (46.9, 51.6)	49.1 (46.9, 51.3)	49.0 (46.3, 51.5)	48.0 (44.8, 50.9)
White Population (%)	60.2 (31.6, 78.1)	69.5 (49.3, 82.2)	70.8 (50.3, 82.7)	56.8 (32.5, 74.6)	30.4 (8.1, 61.6)
Black Population (%)	9.5 (3.2, 33.7)	3.4 (1.5, 7.6)	6.5 (2.8, 16.0)	15.2 (5.7, 35.3)	52.5 (14.0, 86.0)
Median Age	35 (32, 40)	38 (34, 43)	37 (33, 41)	34 (31, 38)	33 (29, 37)
Hispanic Population (%)	15.8 (6.2, 40.6)	11.5 (6.6, 20.6)	18.1 (7.1, 36.4)	27.1 (8.6, 58.5)	13.6 (2.6, 56.2)
Less than High School (%)	8.8 (4.1, 15.4)	4.3 (1.8, 8.9)	5.9 (3.0, 11.6)	10.5 (6.0, 17.1)	14.1 (9.9, 19.7)
Annual Median Household Income ($)	56,042 (38,494, 80,859)	90,082 (68,687, 118,591)	67,623 (54,385, 85,479)	50,253 (40,250, 63,402)	32,181 (25,629, 40,357)

[1] Median (IQR)

© 2025 Chen Z et al. *JAMA Network Open*.

eTable 2. Sensitivity Analysis of Model Performance for Obesity Prevalence Using 2021 CDC PLACES Data

LMEM Model	AIC	BIC	Marginal R2	Conditional R2	Log-Likelihood	Deviance	Compare to	Chi-Square	p-value
**DSE+SDOH**	19643	19719	0.654	0.912	-9809.3	19619	—	—	—
**DSE+SDOH+GSI +GSV**	18856	20005	**0.760**	**0.929**	-9246.8	18494	DSE+SDOH	1125	< .001

DSE: *Demographic and Socioeconomic factors (male, Black, median age, Hispanic, education level below high school, median income);* ***SDOH***: *Social Determinants of Health (Social Vulnerability Index [SVI], Socioeconomic Disadvantage Index [SDI], Area Deprivation Index [ADI]);* ***GSI***: *Google Satellite Imagery-derived environmental features;* ***GSV***: *Google Street View-derived environmental features.*

© 2025 Chen Z et al. *JAMA Network Open*.

eTable 3. Variance Inflation Factor (VIF) Values for Key Demographic, Socioeconomic, and SDOH Predictors

Category	Variable	VIF
**SDOH Indicators**	Socioeconomic Disadvantage Index (SDI)	13.639
	Social Vulnerability Index (SVI)	9.009
	Area Deprivation Index (ADI)	5.419
**Socioeconomic Factors**	Median Household Income	4.898
	% Without High School Education	4.514
**Demographic Factors**	% Hispanic Population	4.198
	% Black Population	3.017
	Median Age	1.901

© 2025 Chen Z et al. *JAMA Network Open*.

eTable 4. Sensitivity Analysis of Model Performance for Obesity Prevalence after removing SDI and SVI.

LMEM Model	AIC	BIC	Marginal R^2	Conditional R^2	Log-Likelihood	Deviance	Test	Chi-Square	p-value
**ADI + SDOH**	18,012	18,076	0.648	0.902	-8,996.0	17,992	—	—	—
**ADI + SDOH + GSI + GSV**	17,240	18,379	**0.755**	**0.922**	-8,441.2	16,882	ADI + SDOH	1,109.6	< .001

© 2025 Chen Z et al. *JAMA Network Open*.
