# Supplementary material for: AI-Enhanced Analysis of Built Environment Imagery and Neighborhood Obesity in US Cities
Source: JAMA Netw Open. 2025 Sep 30;8(9):e2534612. doi: 10.1001/jamanetworkopen.2025.34612 (PMC12485642; doi:10.1001/jamanetworkopen.2025.34612)
Supplement: Supplement 2. — Data Sharing Statement [file jamanetwopen-e2534612-s002.pdf]

Data Sharing Statement

Chen. AI-Enhanced Analysis of Built Environment Imagery and Neighborhood Obesity in US Cities. *JAMA Netw Open*. Published online September 30, 2025. doi:10.1001/jamanetworkopen.2025.34612

Data

Data available: Yes
Data types: Data (not involving human participants), Data dictionary
How to access data: No individual-level participant data were collected in this study. The processed, de-identified census tract–level dataset used for analysis—including data derived from publicly available sources (e.g., CDC PLACES, U.S. Census Bureau, American Community Survey, and Google Street View/Satellite imagery)—will be made available beginning at the time of publication with no end date. Available materials will include the final dataset, data dictionary, codebook, statistical analysis plan, and analysis code. Data will be shared for non-commercial academic research purposes with researchers who submit a methodologically sound proposal. Access will be granted upon approval of the proposal and signing of a data access agreement. Requests should be directed to the lead author Z.C. (zxc738@case.edu) or the corresponding author S.R. (sanjay.rajagopalan@uhhospitals.org) or S.A.K. (sal-kindi@houstonmethodist.org) Data will be shared without investigator support.
When available: With publication

Supporting Documents

Document types: Statistical/analytic code
How to access documents: Requests for statistical/analytic code should be directed to the lead author Z.C. (zxc738@case.edu) or the corresponding author S.R. (sanjay.rajagopalan@uhhospitals.org) or S.A.K. (sal-kindi@houstonmethodist.org) Data will be shared without investigator support.
When available: With publication

Additional Information

Who can access the data: Researchers whose proposed use of the data has been approved
Types of analyses: For a specified purpose
Mechanisms of data availability: After approval of a proposal and without investigator support
